# The lysosomotropic drug LeuLeu-OMe induces lysosome disruption and autophagy-independent cell death in *Trypanosoma brucei*

**DOI:** 10.15698/mic2015.08.217

**Published:** 2015-07-30

**Authors:** Hazel X. Koh, Htay M. Aye, Kevin S. W. Tan, Cynthia Y. He

**Affiliations:** 1 Department of Biological Sciences, National University of Singapore.; 2 Department of Microbiology, National University of Singapore.

**Keywords:** Trypanosoma brucei, LeuLeu-OMe, lysosome, lysosomotropic, necrosis, autophagy

## Abstract

Background: *Trypanosoma brucei *is a blood-borne, protozoan parasite that causes African sleeping sickness in humans and nagana in animals. The current chemotherapy relies on only a handful of drugs that display undesirable toxicity, poor efficacy and drug-resistance. In this study, we explored the use of lysosomotropic drugs to induce bloodstream form *T. brucei* cell death via lysosome destabilization. Methods: We measured drug concentrations that inhibit cell proliferation by 50% (IC<sub>50<sub>) for several compounds, chosen based on their lysosomotropic effects previously reported in *Plasmodium falciparum*. The lysosomal effects and cell death induced by L-leucyl-L-leucyl methyl ester (LeuLeu-OMe) were further analyzed by flow cytometry and immunofluorescence analyses of different lysosomal markers. The effect of autophagy in LeuLeu-OMe-induced lysosome destabilization and cytotoxicity was also investigated in control and autophagy-deficient cells. Results: LeuLeu-OMe was selected for detailed analyses due to its strong inhibitory profile against *T. brucei *with minimal toxicity to human cell lines *in vitro*. Time-dependent immunofluorescence studies confirmed an effect of LeuLeu-OMe on the lysosome. LeuLeu-OMe-induced cytotoxicity was also found to be dependent on the acidic pH of the lysosome. Although an increase in autophagosomes was observed upon LeuLeu-OMe treatment, autophagy was not required for the cell death induced by LeuLeu-OMe. Necrosis appeared to be the main cause of cell death upon LeuLeu-OMe treatment. Conclusions: LeuLeu-OMe is a lysosomotropic agent capable of destabilizing lysosomes and causing necrotic cell death in bloodstream form of *T. brucei*.

## INTRODUCTION

African sleeping sickness (also known as Human African Trypanomiasis, or HAT) is endemic in 36 countries in sub-Saharan Africa, endangering life and economy of ~ 60 million people living in the area 1. The disease has two characteristic stages: the early stage is due to a hemolymphatic infection, and the late stage to an infection of the central nervous system. Clinical manifestation in the early stage includes fever, headache and pains in joints. In the late stage, the manifestations are more severe and symptoms may include personality changes, motor and sensory abnormalities, insomnia, cerebral edema, and coma 2,3. Infection is lethal if left untreated 4.

The causative agents of the disease are parasitic protozoa of the species *Trypanosoma brucei*. They live and multiply in blood and tissue fluids of their mammalian hosts (the bloodstream form) and are transmitted to humans through the bite of an infected tsetse fly of the *Glossina *genus. Though the number of infection cases has dropped significantly during the past years due to a series of control activities, new tools for vector control, diagnosis, and case treatment are needed towards HAT elimination declared by the World Health Organization in 2014 5,6,7. Presently, there are only a handful of drugs available for treating HAT. Some of these drugs are plagued by various problems such as side effects, high cost, acute toxicities, poor oral bioavailability, long treatment, low efficacies, and drug resistance 6. Identification of new targets for the development of anti-trypanosomal drugs is of importance.

Lysosomes are membrane-bounded, acidic organelles containing proteases and other hydrolytic enzymes that are responsible for degradation of macromolecule derived from endocytosis and/or autophagy pathways. They are essential for normal cellular functions and malfunction of lysosomes has been implicated in many human diseases 8,9,10. A large gamut of stimuli such as oxidative stress, ultraviolet exposure and lysosomotropic detergents have been found to induce lysosomal membrane permeabilization in mammalian cells, which is distinctly characterized by the rupture of lysosomal membranes and the translocation of lysosomal components, including enzymes, from the lysosomal lumen to the cytosol. Perhaps the best described is the mechanism of lysosomotropic detergents 11,12,13. These agents are described as basic amphiphilic amines that can attain concentrations several hundred-fold higher within the lysosomes than in the cytosol 14. These amines can freely diffuse across membranes in their uncharged form but becomes trapped in their protonated form when they are localized in acidic vesicles. Accumulation of the protonated form above a certain threshold concentration results in osmotic swelling and acquisition of detergent-like properties which induces lethal lysosomal destabilization and cell death 12. Lysosomotropic agents were shown to trigger a variety of death-associated morphologies ranging from classical apoptosis to necrosis in mammalian cells. The type of cell death depends on the type of lethal stimulus, the extent of lysosomal membrane permeabilization, the amount and the type of enzymes released into the cytoplasm 11.

The lysosomes of parasitic unicellular protozoa, such as *Plasmodium *and Trypanosomatids, share similar properties and functions to those of mammalian cells, and lysosomal membrane permeabilization-induced cell death have been observed and characterized in *Plasmodium *and *Leishmania* parasites 15,16,17,18. Ch’Ng *et al. *18 demonstrated that a group of lysosomotropic compounds including chloroquine (CQ), L-leucyl-L-leucyl methyl ester (LeuLeu-OMe), chlorpromazine (CPZ), promethazine (PMZ), desipramine (DSP) and 4-hydroxytamoxifen (4-HT), all disrupted the digestive vacuole of the malaria parasite, *Plasmodium falciparum*, causing lysosomal membrane permeabilization and triggered downstream programmed cell death pathways such as mitochondria dysregulation and DNA degradation. Among them, LeuLeu-OMe was also shown to target the lysosomal system of intracellular and isolated amastigotes of *Leishmania amazonensis *15. The targets of the esters have been identified as acid-phosphatase-positive megasomes, lysosome-like organelles that are electron-dense. This was supported by the observation that incubation with L-leucine methyl ester (Leu-OMe) resulted in swelling and fusion of megasomes, decreased electron density of the internal contents and the release of acid phosphatase into the medium 16. Adade *et al.* 17 also demonstrated that Leu-OMe was toxic to all three developmental forms of *Trypanosoma cruzi, *targeting in particular acidic compartments in the parasite. They showed a decrease in overall acridine orange fluorescence in the organelles by flow cytometry and a dispersed fluorescence throughout the cell cytoplasm by confocal microscopy. The effect of lysosomtropic drugs is yet to be analyzed in *T. brucei, *the trypanosomatid most accessible to molecular genetic manipulations.

In this study, LeuLeu-OMe-induced lysosomal morphological change was characterized in *T. brucei*. Results indicated that LeuLeu-OMe caused destabilization of the lysosome and necrotic cell death in the parasite. Although autophagosome formation was observed upon LeuLeu-OMe treatment, autophagy did not contribute to LeuLeu-OMe-indued cytotoxicity. These results support lysosomal destabilization as a novel therapeutic approach for future anti-trypanosome drug design.

## RESULTS

### Cytotoxicity of LeuLeu-OMe

Several lysosomotropic compounds including LeuLeu-OMe, CQ, CPZ, PMZ, DSP and 4-HT, have been found to exhibit parasiticidal effects by targeting the vacuolar compartment in *Plasmodium falciparum* 18. To evaluate if similar lysosomotropic effects can be induced in bloodstream form *T. brucei, *cells were incubated with the above-mentioned compounds at various concentrations for 24 h and the cell viability was monitored using flow cytometry system. The IC_50_ values were then calculated for each compound (Table 1) and compared with their IC_50_ against mammalian cells based on published results. Notably, LeuLeu-OMe and CQ both recorded a higher selectivity index (> 5.0) while the other drug compounds were less selective. LeuLeu-OMe, which has the highest selectivity index, was therefore chosen for further studies.

**Table 1:** IC50 of selected lysosomotropic drugs in T. brucei. The inhibitory concentration (IC50) values of 6 lysosomotropic drugs on bloodstream form T. brucei were measured in this study and compared with those reported in mammalian cells (13, 42, 43). *Note that mammalian IC50 values for CQ, CPZ, PMZ, DSP and 4HT were measured on H9c2 rat-cardiomyocyte-derived cells, whereas mammalian IC50 for LeuLeu-OMe was estimated as the concentration observed to induce necrosis in 8 human cell lines including human neuroblastoma cell line SH-SY5Y, human immortalized keratinocytes HaCaT, human adenocarcinoma cell line HeLa, human hepatoma cell line HepG2, human colon carcinoma cell line CaCo-2, and human embryonic kidney fibroblasts HEK293, human breast carcinoma cell line MCF-7 and normal human dermal fibroblasts, NHDF, cytotoxic T lymphocytes and NK cells. LeuLeu-OMe = L-leucyl-L-leucyl methyl ester; CQ = Chloroquine; CPZ = Chlorpromazine; PMZ = Promethazine; DSP = Desipramine; 4HT = 4-hydroxytamoxifen. The IC50 values for T. brucei were shown as mean (95% confidence interval) from >=4 experiments for each drug.

**Drugs**	**IC_50_ for* T. brucei *(µm)**	IC_50_ for mammalian cells* **(µm)**	Selectivity Index (IC_50_ mammalian* / IC_50_* T. brucei*)
**LeuLeu-OMe**	16.8 (14.6-19.4)	125-5000	7.44-298
**CQ**	29.9 (20.5 - 43.8)	148.3	5
**CPZ**	9.21 (7.80-10.9)	20.9	2.27
**PMZ**	13.4 (9.7-18.4)	38.7	2.89
**DSP**	8.8 (6.37-12.2)	10.7	1.22
**4HT**	6.23 (3.02-12.8)	6.6	1.06

**Figure 1:**
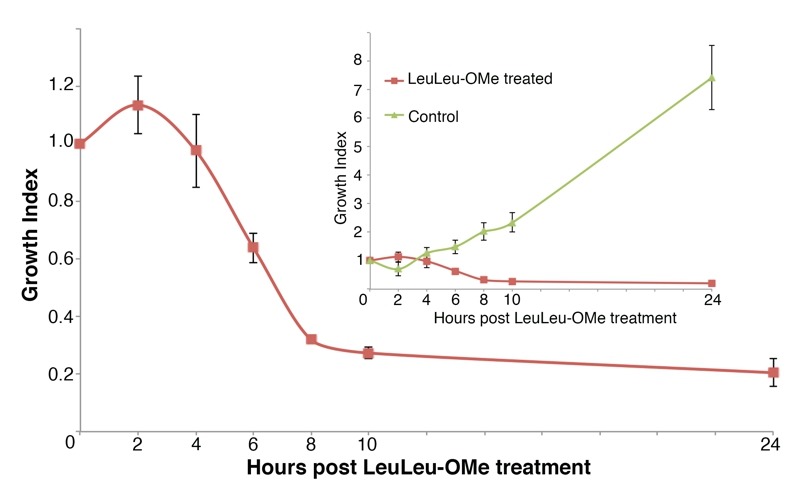
FIGURE 1: Cytotoxicity of LeuLeu-OMe to bloodstream form *T. brucei*. Cells were incubated with or without 30 µM LeuLeu-OMe for up to 24 h. Samples were taken at the indicated time points and monitored for cell growth. Decreased cell number was observed after 2 h of LeuLeu-OMe treatment, while control cells continued to proliferate (see inset for comparison). Growth index was calculated as described in Methods. The results were presented as mean ± SD from 3 independent experiments.

The cytotoxicity of LeuLeu-OMe was further evaluated in cells treated with 30 µM LeuLeu-OMe, which corresponds to the IC_90_ concentration. In this experiment, cell density were monitored every 2 h and the results normalized to cell concentration at t = 0 to obtain growth index (Figure 1). The cells were viable and able to proliferate moderately until 2 h post LeuLeu-OMe treatment, significant cell death was observed between 4 h and 8 h of treatment. As a control, cells with 1% DMSO (solvent for LeuLeu-OMe) added to the cultivation medium, proliferated normally with a doubling time of ~ 8 h (Figure 1, inset). Due to the rapid cell death observed in the first 8 h of LeuLeu-OMe treatment, most subsequent analyses described in this study were restricted to this time period.

### Lysosome destabilizing effects of LeuLeu-OMe

**Figure 2:**
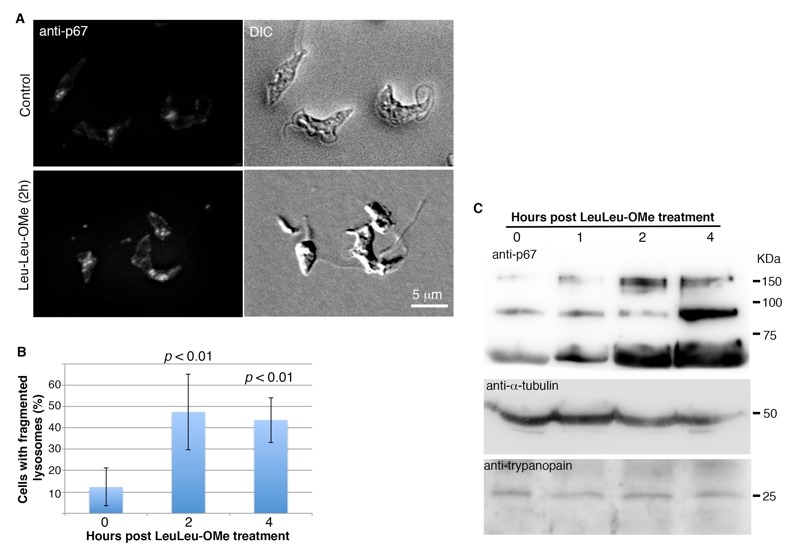
FIGURE 2: Lysosome destabilizing effects of LeuLeu-OMe. **(A)** *T. brucei* cells were incubated with or without 30 µM LeuLeu-OMe, and samples were taken at t = 0 h, 1 h, 2 h and 4 h for immunofluorescence assays with anti-p67. Representative images of control and LeuLeu-OMe-treated cells are maximum intensity projection of serial optical sections through the entire cells. **(B) ** Quantitation of images as shown in (A) revealed an increase in lysosome fragmentation at 2 h and 4 h post Leuleu-OMe treatment. The quantitation results are presented as mean ± SD from 3 independent experiments. **(C) ** Samples treated as described in (A) were also processed for immunoblotting analyses with anti-p67 and anti-trypanopain. Anti-α-tubulin was used as a loading control.

To determine if LeuLeu-OMe has lysosome destabilizing effects in *T. brucei, *p67, an integral lysosome membrane protein, were used to monitor lysosome morphology by immunofluorescence assays during the course of 30 µM LeuLeu-OMe treatment. Most control cells contained one or two major puncta representing lysosomes in different cell cycle stages (Figure 2A), similar to a previous report 19. Upon LeuLeu-OMe treatment, the lysosome staining by p67 became more diffused and fragmented as shown by two-dimensional projection of serial optical sections (sMovie 1 and sMovie 2 in Supplemental Materials). The images taken at t = 0 h, 2 h and 4 h post-LeuLeuOMe treatment were then quantitated, and the percentage of cells containing fragmented lysosomes (i.e. > 2 puncta in each cell) was found to increase from 12% at t = 0, to > 40% at 2 h and 4 h (Figure 2B). Interestingly, the increased lysosmal fragmentation was also accompanied with an increase in p67 expression (Figure 2C). However, the protein level of trypanopain, a lysosome luminal protein, remained unchanged during the course of LeuLeu-OMe treatment. Other membrane bound organelles, including ER, Golgi apparatus, glycosomes and acidocalcisomes, all appeared normal when labeled with anti-BiP, anti-GRASP, anti-SKL and anti-TbVP1 respectively (data not shown).

**Figure 3:**
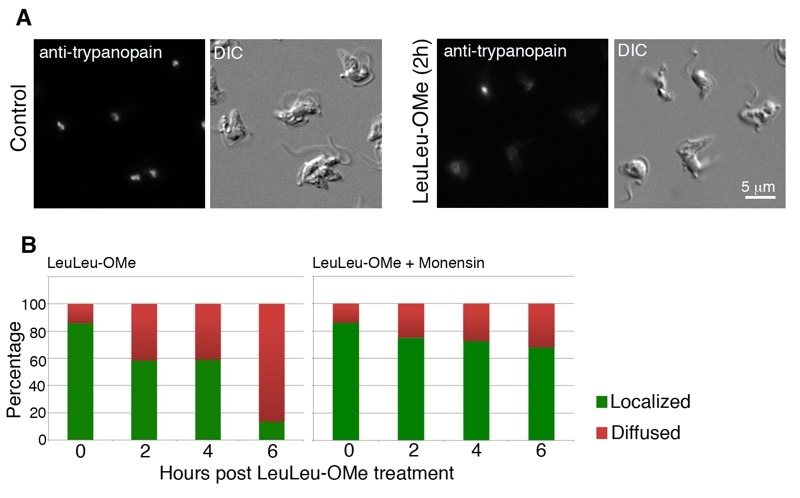
FIGURE 3: LeuLeu-OMe-induced lysosome destabilization revealed by anti-trypanopain staining. **(A) ** Control and LeuLeu-OMe-treated cells were fixed and stained with anti-trypanopain. The staining was localized to one or two puncta in control cells, but became diffused in LeuLeu-OMe-treated cells. Control and drug-treated cells were processed for immunostaining using same conditions. Note that LeuLeu-OMe-treated cells were exposed longer to capture the weaker anti-trypanopain staining in the cytosol. **(B) ** Cells with localized (green) or diffused (red) anti-trypanopain staining were quantitated over the course of LeuLeu-OMe treatment (left), and in cells pre-treated with monensin (right). At least 200 cells were counted for each time point.

To further evaluate lysosome structural integrity, LeuLeu-OMe-treated cells were also labeled with anti-trypanopain. We reasoned that if lysosome membrane integrity was compromised during drug treatment, the staining of the soluble lysosome luminal trypanopain would no longer be confined and may be found in cytosol. Indeed, upon LeuLeu-OMe treatment, more cells were found to contain weak and diffused anti-trypanopain staining distributed throughout the cell (Figure 3A). As the expression level of trypanopain did not change during the course of LeuLeu-OMe treatment (see Figure 2C), the change from localized to diffused staining suggested release of lysosomal trypanopain into the cytosol, providing a convenient marker to evaluate lysosome structure integrity during LeuLeu-OMe treatment and other conditions (Figure 3B).

**Figure 4:**
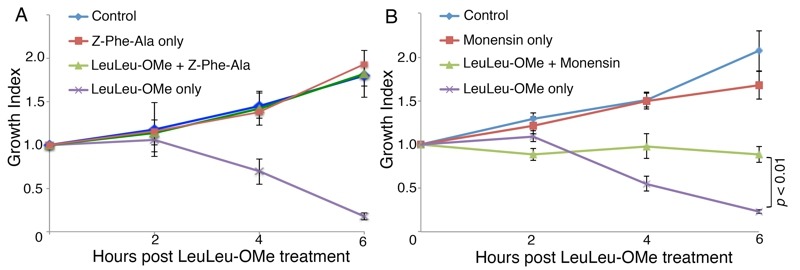
FIGURE 4: Treatment with a cathepsin inhibitor or monensin reduces LeuLeu-OMe-induced cell death. **(A) ** Cells were treated with 10 µM Z-Phe-Ala or 30 µM LeuLeu-OMe, alone or in combination. **(B) ** Cells pre-treated with 100 nM monensin for 30 min was then cultivated in the presence or absence of 30 µM LeuLeu-OMe. While monensin treatment alone had no observable effects on parasite growth (compared to control), monensin treatment significantly reduced LeuLeu-OMe-induced cytotoxicity. Results were presented as mean ± SD from 3 independent experiments. p value for the samples treated with LeuLeu-OMe only or LeuLeu-OMe + monensin at 6 h time point is indicated next to the bracket.

Due to disruption of lysosomal integrity, LeuLeu-induced cell death is likely caused by the release of lysosomal cathepsins into the cytosol. To test this possibility, LeuLeu-OMe-induced cell death was further evaluated in the presence of cathepsin inhibitor Z-Phe-Ala 20. Treatment of cells with Z-Phe-Ala alone, up to 40 µM, had little effect on parasite growth by 9h. Prolonged treatment (24h) showed a dose-dependent inhibition of cell proliferation (Figure S1). Treatment of cells with LeuLeu-OMe together with 10 µM Z-Phe-Ala complete rescued the rapid cell death induced by LeuLeu-OMe at IC_90_ (Figure 4A), supporting a role of cathepsins in LeuLeu-OMe-induced cell death.

### Lysosome acidity is required for LeuLeu-OMe cytotoxicity

Lysosomotropic detergents are known for their ability to accumulate in vesicles that possess an acidic interior (low pH). Raising the pH of the lysosome has been shown to decrease the uptake of these detergents, indicating the importance of a low pH for their entry into lysosomes 12. To verify if the acidic pH of lysosomes was required for LeuLeu-OMe cytotoxicity in *T. brucei, *cells were pre-incubated with 100 nM monensin (an ionphore and a Na+/H+ antiporter) for 30 min, which was sufficient to neutralize the acid pH and inhibit lysotracker accumulation in lysosomes (data not shown). At this concentration, monensin alone had no observable effects on cell viability, but significantly reduced cytotoxicity and lysosome destabilizing effects in LeuLeu-OMe treated cells (Figure 3B and Figure 4B). These results are consistent with the effects of LeuLeu-OMe on lysosomes, and supported a role of acidic pH in LeuLeu-OMe action.

### LeuLeu-OMe-induced cell death in *T. brucei*

**Figure 5:**
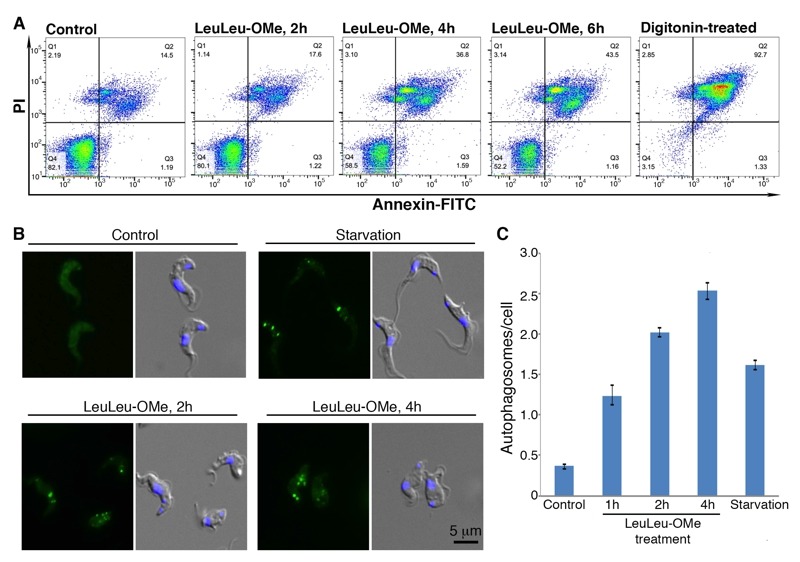
FIGURE 5: LeuLeu-OMe induces necrotic cell death and autophagy. **(A) ** Cells treated with 30 µM LeuLeu-OMe were fixed and stained with PI and annexin-FITC, and analyzed by flow cytometry. Digitonin-permeabilized cells were used as a positive control for the stains. **(B, C) ** Cell stably expressing YFP-TbATG8.2 were cultivated in medium with or without 30 µM LeuLeu-OMe. Autophagosome formation was monitored by relocalization of YFP-TbATG8.2 from a cytoplasmic distribution in control cells to a punctate structures using fluorescence microscopy. Autophagosome formation was quantitated and the results are shown as mean ± SD from 3 independent experiments. Cells starved in cytomix for 2 h were used as a positive control.

To further investigate how LeuLeu-OMe induced *T. brucei *cell death, control and cells treated with 30 µM LeuLeu-OMe were evaluated for necrotic and apoptotic cell death, using propidium iodide (PI) and Annexin-FITC stains. Cells positively stained for both PI and Annexin-FITC were found to increase from 14.4% in the control population to 17.5% at 2 h, 36.4% at 4 h and 43.3% at 6 h of LeuLeu-OMe treatment (Figure 5A), indicating an increase of necrotic cells in a time-dependent fashion. Increase in apoptotic cells (positively stained with Annexin-FITC but negative for PI) was not observed during LeuLeu-OMe treatment. To rule out the possibility that the lack of apoptosis may be due to rapid cell killing by high concentration of LeuLeu-OMe (30 µM, which corresponded to IC_90_), the above same experiments were repeated in cells treated with 16 µM LeuLeu-OMe, which corresponded to IC_50_ (Figure S2A). While cell death was slowed at the lower drug concentration, we still did not observe an increase in apoptotic cells.

Autophagy-dependant cell death has been previously reported in many protozoan pathogens, including *T. brucei *and *Plasmodium*, upon drug treatment and starvation stresses 21,22,23,24. To evaluate if autophagy may be involved in LeuLeu-OMe-induced cell death in *T. brucei, *cells stably expressing YFP-TbATG8.2 were incubated with 30 µM (IC_90_) LeuLeu-OMe, and autophagosome formation was monitored by fluorescence microscopy. In control cells, YFP::TbATG8.2 exhibited a cytoplasmic distribution, similar to previously observed in procyclic *T. brucei* 21. Upon starvation stress, YFP::TbATG8.2 relocated to autophagosomes that appear as punctate structures throughout the cell (Figure 5B). A steady increase in autophagosome numbers was observed in LeuLeu-OMe-treated cells in a time-dependant fashion (Figure 5C).

**Figure 6:**
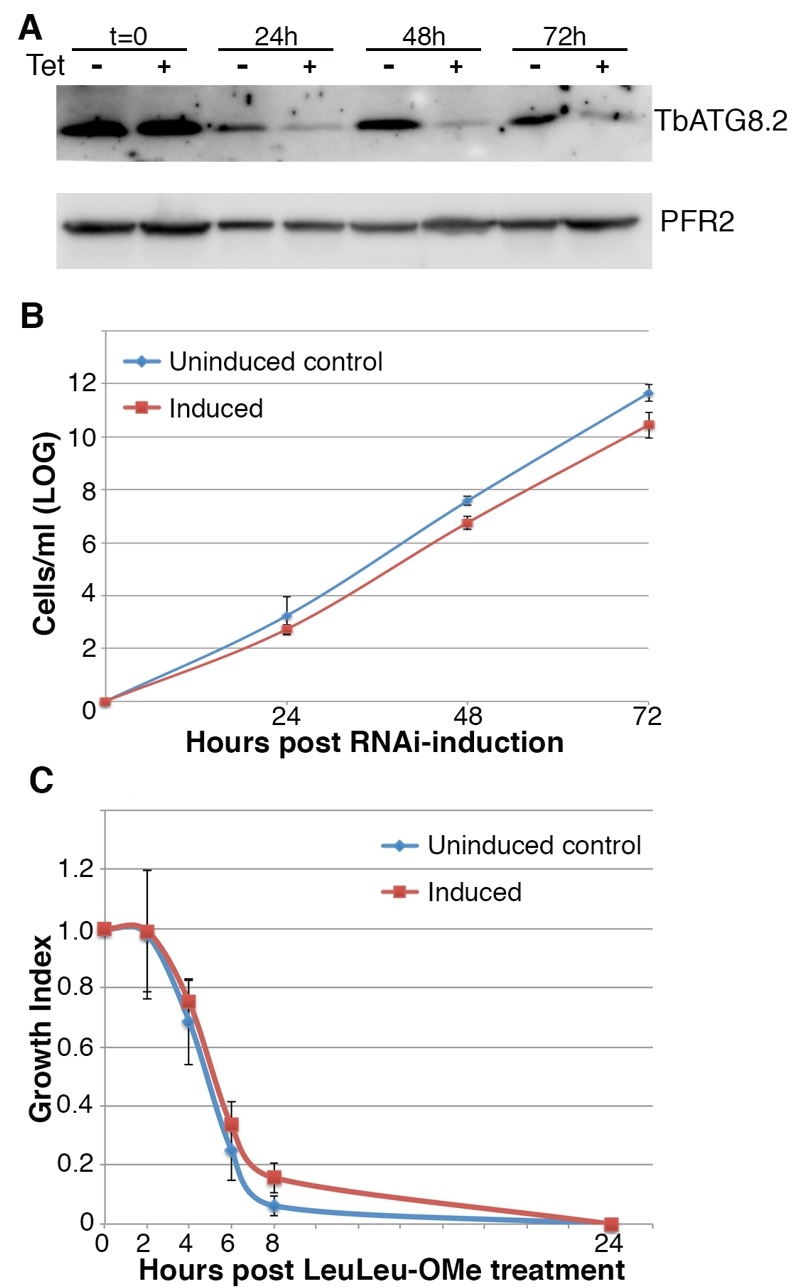
FIGURE 6. Autophagy is not required for LeuLeu-OMe-induced cell death. **(A, B) ** Cells were stably transfected with pZJM-TbAtg8.1/8.2 for inheritable and inducible RNAi of TbATG8.1 and TbATG8.2 expression. The cells were induced with tetracycline, and samples were taken at t = 0 h, 24 h, 48 h and 72 h post-induction for immunoblots (A) and cell counting (B). Anti-TbATG8.2 antibody was used to monitor TbAtg8.1/8.2-RNAi efficiency, and anti-PFR2 (a component of paraflagellar rod) was used as a loading control. **( C) ** Cells induced for TbAtg8.1/8.2-RNAi or not were also treated with 30 µM LeuLeu-OMe, and cell growth monitored as described in Figure 1. Results shown in (B) and (C) are presented as mean ± SD from 3 independent experiments.

To determine the effect of autophagy on LeuLeu-OMe-induced cell death, a stable RNAi cell line targeting both Atg8 homologs in *T. brucei, *TbAtg8.1 and TbAtg 8.2 21, was constructed. Efficient RNAi was shown by immunoblotting with anti-ATG8.2 antibody (Figure 6A), and TbAtg8.1/8.2 double RNAi had little effect on cell proliferation under normal growth conditions (Figure 6B). Upon treatment with 30 µM (IC_90_) or 16 µM (IC_50_) LeuLeu-OMe, the uninduced control and cells induced for TbAtg8.1/8.2-RNAi did not exhibit significant differences in cell growth (Figure 6C and supplementary Figure S2B), suggesting that autophagy did not play a major role in LeuLeu-OMe-induced cell death.

## DISCUSSION

Lysosomes have long been deemed as a waste bin where macromolecules derived from outside of the cell through endocytosis or from within the cell through autophagy are degraded and recycled. Full of proteases and other hydrolytic enzymes, the lysosome is also considered a ‘dangerous’ place. While the presence of lysosome associated membrane proteins such as LAMP1 and LAMP2 can protect lysosome itself from the hydrolytic enzymes 25, damage to lysosome integrity may allow these enzymes to leak into the cytosol and cause unintended damage to other cellular components. This damaging potential of lysosomes have long been noted and been explored as a mechanism to induce cell death 11. In particular, the action of lysosomotropic detergents has been well documented in mammalian cells ever since the 1980s, when lysosomotropic compounds were chemically synthesized to mimic the lysosome accumulation and lysosomotropic properties of amines 26. Cell death induced by lysosomotropic activities is of particular interest and offers a potential new drug target in protozoan parasites, which are single-celled eukaryotic pathogens living inside mammalian hosts.

We have previously reported evidence that several lysosomotropic drugs can cause digestive vacuole (similar to lysosomes) destabilization in the malaria parasite, *P. falciparum, *and that digestive vacuolar membrane permeabilization triggers the program cell death pathway 18. In this study, we have extended this work to another important human and animal parasite *T. brucei. *

We have focused on a well-characterized lysosomotropic detergent, LeuLeu-OMe, which shows potent inhibition of *T. brucei* with IC_50_ in the micromolar range and is the most selective of 6 lysosomotropic agents tested in this study. Similar to that reported in mammalian cells as well as other organisms, LeuLeu-OMe treatment led to lysosome fragmentation and release of lysosomal lumenal protein trypanopain. The use of two different lysosomal markers, p67, which is a LAMP-like lysosome membrane glycoprotein 27, and trypanopain, which is a major cathepsin-L type protease 28, provided a highly reproducible and reliable method to examine lysosome integrity. It is interesting to note that the levels of different p67 glycosylation forms increased significantly during the course of LeuLeu-OMe treatment. This up-regulation may be due to the cell’s compensatory effect in response to the lysosome destabilizing activity of LeuLeu-OMe, considering the role of p67 in lysosome structure maintenance 27. In contrast, trypanopain level remained unchanged during the treatment, though its cellular distribution changed from a localized pattern in control cells, to a diffused distribution in LeuLeu-OMe treated cells, consistent with a role of LeuLeu-OMe in compromising lysosome integrity.

Both necrotic and apoptotic cell death have been described following treatment with lysosomotropic drugs, and the exact type of cell death is likely dependent on the extent of lysosome destabilization and thus influencing the amount and type of enzymes that are released into the cytoplasm 11. An example is the sphingolipid sphingosine, which is a lipophilic weak base. Upon protonation, sphingosine accumulates within acidic compartments where it may act as a detergent. Limited doses of sphingosine induce a cascade of lethal events including lysosomal membrane permeabilization, caspase activation, as well as the dissipation of the mitochondrial membrane potential. On the other hand, high doses rapidly cause lysosomal rupture culminating in rapid necrosis 29. Other examples include the lysosomotropic detergents *N*-dodecylimidazole and *O*-methyl-serine-dodecylamide hydrochloride, both induce lysosome destabilization followed by caspase-3-dependent apoptosis at low doses and necrosis at high doses 30,31. It is unlikely that the lack of apoptosis was due to rapid killing by LeuLeu-OMe, as lower dose of LeuLeu-OMe that killed the cells at slower kinetics did not lead to apoptosis either. Notably, the presence of apoptosis in *T. brucei *and other protozoan parasites has long been controversial. The lack of key apoptotic molecules such as caspases in the parasite genomes and inconsistency in detection of apoptotic phenotypes suggest that these organisms do not possess the regulated apoptosis pathway as those described in higher eukaryotes 32.

In the recent years, the presence of a conserved autophagy pathway and its molecular machineries has been demonstrated in *T. brucei *and many other protozoan parasites. Although the physiological trigger and function of autophagy in these single-celled organisms remains to be experimentally tested, a possible role of autophagy in cell death has been proposed based on molecular evidence in *T. brucei *and *Toxoplasma gondii* 21,24. It is therefore interesting to note in our study that a significant increase in autophagosome numbers was found upon LeuLeu-OMe treatment, though depletion of autophagy had little effects on the progress of cell death. The increased autophagosome formation observed during LeuLeu-OMe may represent either true autophagy induction, or inhibited autophagosome fusion to the damaged lysosomes. In either case, as lysosome functions, which are the final steps of the autophagy pathway, are inhibited by LeuLeu-OMes, any downstream function of autophagy would also be compromised. This may provide an explanation for the lack of a role for autophagy in LeuLeu-OMe induced cell death.

While neither apoptosis nor autophagy had a role in LeuLeu-OMe-induced cell death, nonspecific action of released lysosomal cathepsins in cytosol following lysosomal disintegration is likely the main cause of cell death. Consistent with this, co-treatment of cells with a cathepsin inhibitor completely reversed the rapid cell death induced by LeuLeu-OMe.

LeuLeu-OMe is a 2-amino acid compound known to have immunosuppressive activity. The drug has been commonly used to decrease the incidence of graft versus host disease (GVHD) via cytotoxic cell depletion *ex vivo* following blood transplant in animal models such as mice and dogs 33,34. LeuLeu-OMe has also been effective in preventing GVHD in humans 35,36. However, LeuLeu-OMe has never been directly administered into animals or humans, and thus its immunosuppressive activity has not been validated in animals. Furthermore, necrotic cell death is not the preferred mode of killing because it might cause inflammation. However, LeuLeu-OMe was the most selective of the 6 lysosomotropic drugs tested in this study. With its anti-trypanosome mechanisms more extensively studied, structure activity relationship can be better explored to optimize leads with better efficacy and tolerability profiles as a lysosome-destabilizing agent.

## MATERIALS AND METHODS

### Cell lines and culture

*Bloodstream form T. b. brucei* single marker cell line 37, a derivative of the 427 strain, was maintained at 37°C, 5% CO_2_, and > 90% humidity in HMI-9 medium supplemented with 10% (v/v) fetal bovine serum (Hyclone). Cells were sub-cultured by diluting into fresh medium every 24 h to maintain exponential growth (with cell density between 1 x 10^5^ and 1 x 10^6^ cells/ml).

### Plasmid construction and transfection

A pZJM construct previously described 21 was used for inducible RNAi of both TbAtg8.1 and TbAtg8.2. Stable RNAi cell lines were generated by electroporating the pZJM-Atg8.1/8.2 vector into the bloodstream form cells using an Amaxa Nucleofector with programme X-001 (Lonza), and selection with 4 µg/ml phleomycin.

### Inducible RNAi

To monitor the proliferation of TbAtg8.1/8.2-RNAi cells, 1 µg/ml tetracycline (Sigma) was added to induce RNAi. Cell density was measured every 24 h using a haemocytometer. The uninduced cells were used as a negative control. During the course of measurements, the cells were maintained in exponential growth phase at a density of 1 x 10^5^ to 1 x 10^6^/ml by dilution with fresh medium with or without tetracycline. For western blot analyses, protein samples (from 1 x 10^7^ cells) were fractionated by 12% sodium dodecyl sulphate polyacrylamide gel electrophoresis (SDS-PAGE). Following transfer, the immunoblots were probed with anti-Atg8.2 antibody (1/1000 dilution) 21. The same blots were also probed with anti-PFR2 38 as loading controls.

### Drug preparations

Stock solution of 4-hydroxytamoxifen (4-HT) (Sigma) was dissolved in ethanol and stored at -20°C. Stock solutions of chloroquine diphosphate (CQ), desipramine (DSP), chlorpromazine (CPZ) and promethazine (PMZ) (all from Sigma), were prepared fresh before each experiment, by dissolving in phosphate buffered saline (PBS) and filter sterilization. Stock solutions of L-leucyl-L-leucyl methyl ester (Sigma) was prepared freshly before each experiment, by dissolving in DMSO. Stock solution of Z-Phe-Ala fluoromethyl ketone (Z-Phe-Ala; Sigma) was prepared in DMSO. Vehicle controls consisted of equivalent amounts, all < 1%, of DMSO (LeuLeu-OMe), PBS (CQ, CPZ, PMZ) and ethanol (4-HT). Monensin (Sigma) was prepared at a final concentration of 100 nM in ethanol. The concentration of monensin was optimized to be the lowest concentration that inhibited lysotracker accumulation in lysosomes, yet did not obviously affect cell growth after 6 hours (data not shown).

### IC_50_ measurements 

Parasite cultures diluted with fresh medium to 10^5 ^cells/ml were incubated with various concentrations of test substances for 24 hours in a 96 well microtiter plate in a final volume of 200 μl. Serial dilutions were made on each drug to create working solutions. The drug concentration range used for LeuLeu-OMe is 1 nM - 1 mM, CQ at 1 nM - 3 mM, CPZ at 1 nM - 100 μM, PMZ at 1 nM - 200 μM, DSP at 1 nM - 250 μM and 4-HT at 1 nM - 100 μM. To determine cell viability at the 24 h time point, cell density was measured using a Guava® flow cytometry system (Millipore, USA). Solvent controls contained DMSO, PBS or ethanol that were diluted in cell cultures to give final concentrations not exceeding 1%. No less than four independent experiments were performed for each data set. Plotting of the dose-response curve as well as the computation of the IC_50_ and IC_90 _(drug concentration that inhibits cell growth by 90%) were performed with GraphPad Prism 5 demo version using a four-parameter logistic curve (variable slope). The selectivity index (SI) values were calculated using the ratio: SI= IC_50_ for mammalian cell/IC_50_ for *T. brucei*

### Growth index 

To monitor cytotoxicity by LeuLeu-OMe and other treatments, cell samples taken at various time points of the treatments were counted using a haemocytometer. Growth index was calculated using the formula below:

Growth index = Cell density at a time point / cell density at t=0

### PI/Annexin assay

To monitor LeuLeu-OMe-incuded cell death, LeuLeu-OMe-treated (30 µM) and untreated parasites were washed and resuspended in 0.5 ml binding buffer (10 mM HEPES, 140 mM NaCl, 2.5 mM CaCl_2_, 10 mM glucose, pH 7.4) and then incubated for 15 min at room temperature with propidium iodide (PI, 5 µg/ml) and annexin V-FITC (0.75 µg/ml). Cells were then analyzed by a Becton-Dickinson LSR Fortessa flow cytometry system (BD Biosciences). Cells permeabilized with 6 µM digitonin for 5 min were used as positive control for PI and annexin V dyes. Compensation of spectral overlap was performed using single-stained control cells with the FlowJo software.

### Fluorescence microscopy

Parasite cultures diluted with fresh medium to approximately 10^5 ^cells/ml were incubated with 30 µM of LeuLeu-Ome. Samples of control and drug treated cells were taken at t = 0 h, 2 h, 4 h and 6 h for immunofluorescence analyses. The samples were fixed in situ with 4% paraformaldehyde for 15 min, washed twice with PBS, attached to poly-L-lysine coverslips, permeabilized in 0.125% Triton X-100 (w/v in PBS) for 15 min, blocked with 3% BSA (w/v in PBS) for 1 hour and then processed for antibody labelling. Antibodies directed against trypanopain, a soluble cysteine cathepsin in the lysosome lumen 28 or p67, a lysosomal membrane protein 39 were used as lysosome markers. Anti-GRASP, anti-SKL, anti-VP1 and anti-BiP were used to label the Golgi 40, the glycosomes 41, the acidocalcisomes 42, and the endoplasmic reticulum (ER) 43, respectively. To image lysosomes, Z-stack images (a total of nine images/stack, with step size of 0.3 µm) were acquired over the entire depth of the cells containing lysosome staining, using Observer Z1 (Zeiss, Germany) equipped with a 63 X NA1.4 objective and a CoolSNAP HQ^2^ CCD camera (Photometrics). Images were processed with ImageJ and Adobe Photoshop.

### Autophagosome formation in YFP:TbAtg8.2 cells

YFP:TbATG8.2 fusion as described previously 21 was subcloned in pHD1034 vector, which allows protein overexpression in the bloodstream form parasites. Stable clones were obtained through selection with 1 µg/ml puromycin. To monitor autophagosome formation, YFP:TbATG8.2 cells were diluted with fresh medium to approximately 10^5 ^cells/ml in the presence or absence of 30 µM LeuLeu-OMe. Cell samples were taken at 1 h, 2 h and 4 h post drug treatments, fixed in situ with 4% paraformaldehyde for 15 min, washed twice with PBS and attached to poly-L-lysine coverslips. Starvation control contained cells incubated with cytomix (2 mM EGTA, 120 mM KCl, 0.15 mM CaCl_2, _10 mM K_2_HPO_4_/KH_2_PO_4, _25 mM HEPES, 5 mM MgCl_2_.6H_2_O, 0.5% Glucose, 100 μg/ml BSA, 1 mM Hypoxanthine, pH 7.6) at 37°C for 2 hours. At least three independent experiments were performed for each condition.

### Statistics

The statistical significance of results from experimental groups in comparison with control groups was determined by the Student’s *t* test. 2 sample equal variance and *p* < 0.05 was considered to be statistically significant.

## SUPPLEMENTAL MATERIAL

Click here for supplemental data file.

All supplemental data for this article are also available online at http://microbialcell.com/researcharticles/the-lysosomotropic-drug-leuleu-ome-induces-lysosome-disruption-and-autophagy-independent-cell-death-in-trypanosoma-brucei/.
