# Supplementary material for: The lysosomotropic drug LeuLeu-OMe induces lysosome disruption and autophagy-independent cell death in Trypanosoma brucei
Source: Microb Cell. 2015 Jul 30;2(8):288–98. doi: 10.15698/mic2015.08.217 (PMC5349101; doi:10.15698/mic2015.08.217)
Supplement: Supplementary file 1 [file mic-02-288-s01.pdf]

The lysosomotropic drug LeuLeu-OMe induces lysosome disruption and autophagy-independent cell death in *Trypanosoma brucei*

Hazel Xinyu Koh[1,2], Htay Mon Aye[1], Kevin S. W. Tan[2]* and Cynthia Y. He[1]*

[1] Department of Biological Sciences, National University of Singapore
[2] Department of Microbiology, National University of Singapore

*Co-corresponding authors:
kevin_tan@nuhs.edu.sg; dbshyc@nus.edu.sg

Running title: LeuLeu-OMe-mediated lysosme destabilization and cell death in *T. brucei*

Supplemental Figures and Videos:

Figure S1. Inhibitory effects of Z-Phe-Ala on *T. brucei* blood stream form cell growth. Cells were incubated with 10, 20, 40 or 100 μM Z-Phe-Ala, which is a potent cathepsin inhibitor. Cell growth at 3h, 6h, 9h and 24h post drug treatment was monitored as described in Figure 1. While an inhibitory effect was observed at 24h at all drug concentrations tested, little inhibitory effect was observed up to 9h for cells treated with 10 or 20 μM Z-Phe-Ala. 10 μM Z-Phe-Ala was therefore used for the subsequent experiments shown in Figure 4A.

Figure S2. Cell death induced by LeuLeu-OMe at IC50. (A) Cells treated with 16 μM LeuLeu-OMe (IC50) were fixed and stained with PI and annexin-FITC, and analyzed by flow cytometry. Digitonin-permeablized cells were used as positive control for the stains. **(B)** Cells induced for TbAtg8.1/8.2-RNAi or not were also treated with 16 μM LeuLeu-OMe. Results are presented as mean±SD from 3 independent experiments.

Movie S1. Serial optical sections through control *T. brucei* cells, fixed and stained with anti-p67. Images were acquired at 0.3 μm steps through the entire cells, deconvolved and shown as Quicktime movie.

Movie S2. Serial optical sections through *T. brucei* cells treated with 30μM LeuLeu-OMe for 2hr, fixed and stained with anti-p67. Images were acquired at 0.3μm steps through the entire cells, deconvolved and shown as Quicktime movie.

Fig. S1

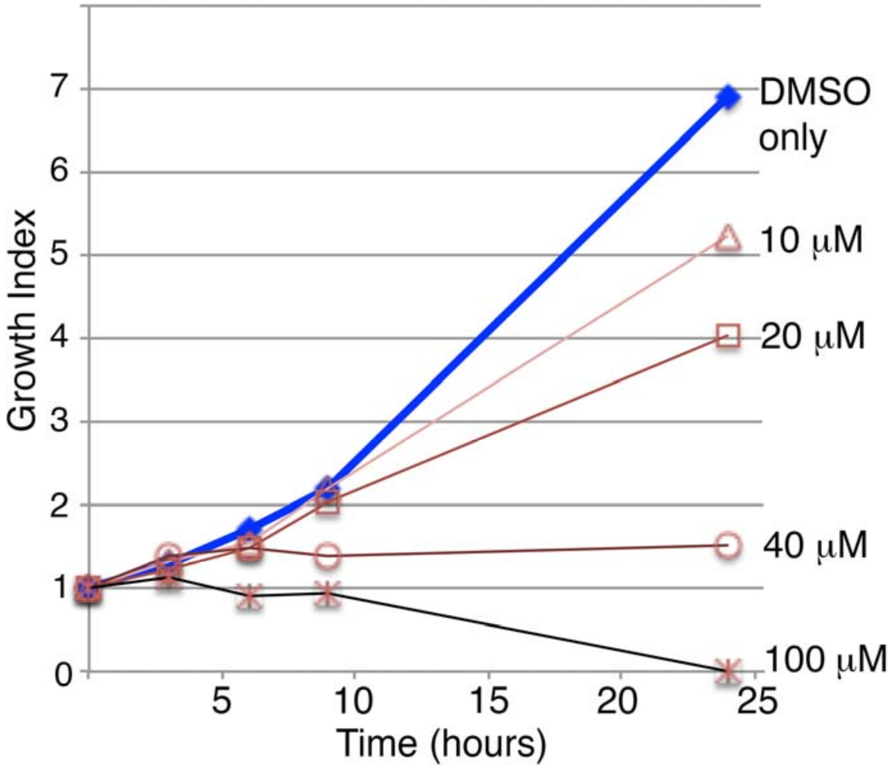

Growth Index
0
1
2
3
4
5
6
7
5
10
15
20
25
Time (hours)
DMSO only
10 μM
20 μM
40 μM
100 μM

Fig. S2

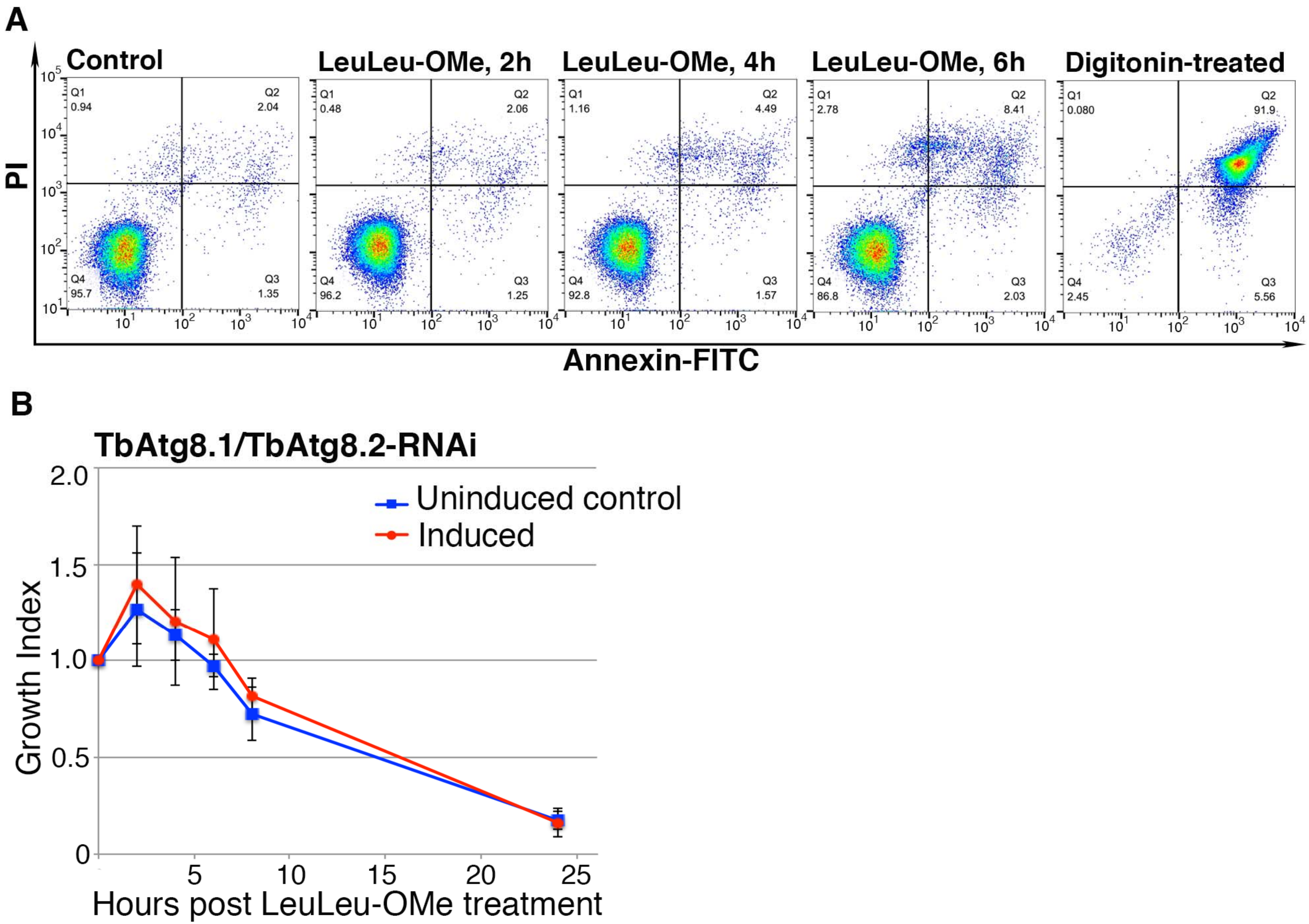
A
Control
LeuLeu-OMe, 2h
LeuLeu-OMe, 4h
LeuLeu-OMe, 6h
Digitonin-treated
PI
Annexin-FITC
Q1 0.94
Q2 2.04
Q4 95.7
Q3 1.35
Q1 0.48
Q2 2.06
Q4 96.2
Q3 1.25
Q1 1.16
Q2 4.49
Q4 92.8
Q3 1.57
Q1 2.78
Q2 8.41
Q4 86.8
Q3 2.03
Q1 0.080
Q2 91.9
Q4 2.45
Q3 5.56
B
TbAtg8.1/TbAtg8.2-RNAi
Uninduced control
Induced
Growth Index
Hours post LeuLeu-OMe treatment
